# Cerebellar cognitive affective syndrome after acute cerebellar stroke

**DOI:** 10.3389/fneur.2022.906293

**Published:** 2022-08-11

**Authors:** Anissa Abderrakib, Noemie Ligot, Gilles Naeije

**Affiliations:** Department of Neurology, CUB Hôpital Erasme, Université libre de Bruxelles (ULB), Brussels, Belgium

**Keywords:** cerebellar cognitive affective syndrome, cerebellar stroke, crossed cerebellar diaschisis, stroke, cognition, prognosis

## Abstract

**Introduction:**

The cerebellum modulates both motor and cognitive behaviors, and a cerebellar cognitive affective syndrome (CCAS) was described after a cerebellar stroke in 1998. Yet, a CCAS is seldom sought for, due to a lack of practical screening scales. Therefore, we aimed at assessing both the prevalence of CCAS after cerebellar acute vascular lesion and the yield of the CCAS-Scale (CCAS-S) in an acute stroke setting.

**Materials and methods:**

All patients admitted between January 2020 and January 2022 with acute onset of a cerebellar ischemic or haemorrhagic first stroke at the CUB-Hôpital Erasme and who could be evaluated by the CCAS-S within a week of symptom onset were included.

**Results:**

Cerebellar acute vascular lesion occurred in 25/1,580 patients. All patients could complete the CCAS-S. A definite CCAS was evidenced in 21/25 patients. Patients failed 5.2 ± 2.12 items out of 8 and had a mean raw score of 68.2 ± 21.3 (normal values 82–120). Most failed items of the CCAS-S were related to verbal fluency, attention, and working memory.

**Conclusion:**

A definite CCAS is present in almost all patients with acute cerebellar vascular lesions. CCAS is efficiently assessed by CCAS-S at bedside tests in acute stroke settings. The magnitude of CCAS likely reflects a cerebello-cortical diaschisis.

## Introduction

Acute vascular cerebellar lesions are proportionally rare and account for 2–3% of all strokes (1, 2). Most cerebellar strokes are considered relatively mild due to low initial National Institutes of Health Stroke Scale (NIHSS) score (3) and mostly favorable outcomes: 70% of patients are deemed fully independent after a cerebellar stroke when consciousness is not impaired at presentation (2, 4, 5). Still, the functional consequences of cerebellar vascular impairment might be underestimated. In the human central nervous system (CNS), the cerebellum hosts four times more neurons than the neocortex and displays with the prefrontal cortex the main relative increase in sapiens' brain neurons compared with other mammals (6, 7). The ratio of cerebellar to neocortical neurons and the cerebellar cortical surface is, furthermore, substantially increased from big apes to humans (6, 8, 9). Historically associated with movement control, the cerebellum is now recognized to play an important role in perceptual and cognitive processes (10, 11). This expansion is one of the explanations for the human brain's higher cognitive performance. The interplay between neocortical areas and the cerebellum occurs thanks to dense reciprocal connections with efferent cerebellar dentato-thalamo-cortical tracts that modulate a wide array of neocortical areas, which in turn are connected back to the cerebellum through cortico-ponto-cerebellar tracts (12–16). The function of the cerebello-cortical loops (CCLs) is to increase the accuracy of both motor and cognitive behaviors (17). Clinically, impairment of the CCL through acute or chronic cerebellar disorders leads to both motor and thought dysmetria (18–20). While motor dysmetria after cerebellar stroke was already well described by Holmes (21), the impact of cerebellar dysfunction on cognitive processes was only outlined two decades ago by Jeremy Schmahmann. In his seminal series, Jeremy Schmahmann described a cohort of twenty patients with cerebellar disorders, thirteen of whom had an acute vascular lesion (22), and various cognitive impairments that included language, emotional regulation, memory, attention, visuospatial, and executive function (22, 23). He coined the cognitive profile associated with cerebellar lesion as the *cerebellar cognitive* affective syndrome (CCAS). This association between cognitive dysfunction and cerebellar disorders was confirmed in several studies [for a meta-analysis, refer to the example in Ahmadian et al. (24)]. However, the cognitive disorders associated with cerebellar pathology are seldom systematically studied due to the extensive and lengthy neuropsychological test batteries (over 90 min in many of the studies) that were, until recently, required to highlight a CCAS. In 2018, a CCAS screening and follow-up scale (CCAS-S) was developed, based on the paper and pencil neuropsychological tests, that could most efficiently single out individuals with cerebellar cognitive disorders from healthy individuals (23). The CCAS-S allows <10 min to provide evidence for a CCAS in patients with cerebellar disorders (23). To date, the CCAS-S has not been used in patients with acute vascular cerebellar disorders.

The aim of this study was therefore to (i) determine the prevalence of a CCAS in a cohort of patients with acute vascular cerebellar lesion and (ii) assess the practicability of the CCAS-S in the context of acute stroke.

## Subjects and methods

### Population

The studied population is derived from the stroke registry of the Erasmus Hospital in Brussels (Belgium) where all cases of acute stroke are recorded since January 2015 (25, 26). Our analysis included patients admitted between January 2020 and January 2022 who had an acute onset cerebellar ischaemic or haemorrhagic stroke and for whom a CCAS-S was performed within 1 week of symptom onset.

### Acute stroke care and clinical evaluation

Acute stroke management and care followed the European Stroke Organization guidelines and are detailed in Elands et al. (25) and Jodaitis et al. (27). Stroke initial severity was evaluated by the National Institutes of Health Stroke Scale (NIHSS) score at admission. CCAS was evaluated using the CCAS-S. The CCAS-S is composed of 10 items: a semantic fluency task, a phonemic fluency task, a verbal category switching task, a forward digit span, a backward digit span, a cube drawing task, a verbal registration task, a verbal similarities task, a Go No-Go task, and an affect evaluation (23). A raw score is obtained for each task, with a minimum passing score. The number of failed tests determines the likelihood that the subject has CCAS: three or more failed tasks make a definite CCAS, two probable CCAS, and one possible CCAS. The raw score ranges from 82 (sum of minimum passing scores for each item on the scale) to 120 (sum of maximum scores for each item) is not diagnostic but provides quantitative values in each task that can be used for longitudinal follow-up as patients can have definite CCAS (three failed test items) with a total raw score that falls in the 82–120 range. Subjects without CCAS are not expected to fail any task (23). The French translation of the “A” version of the CCAS-S (23) was used in this study.

## Results

### Population

**Figure 1:**
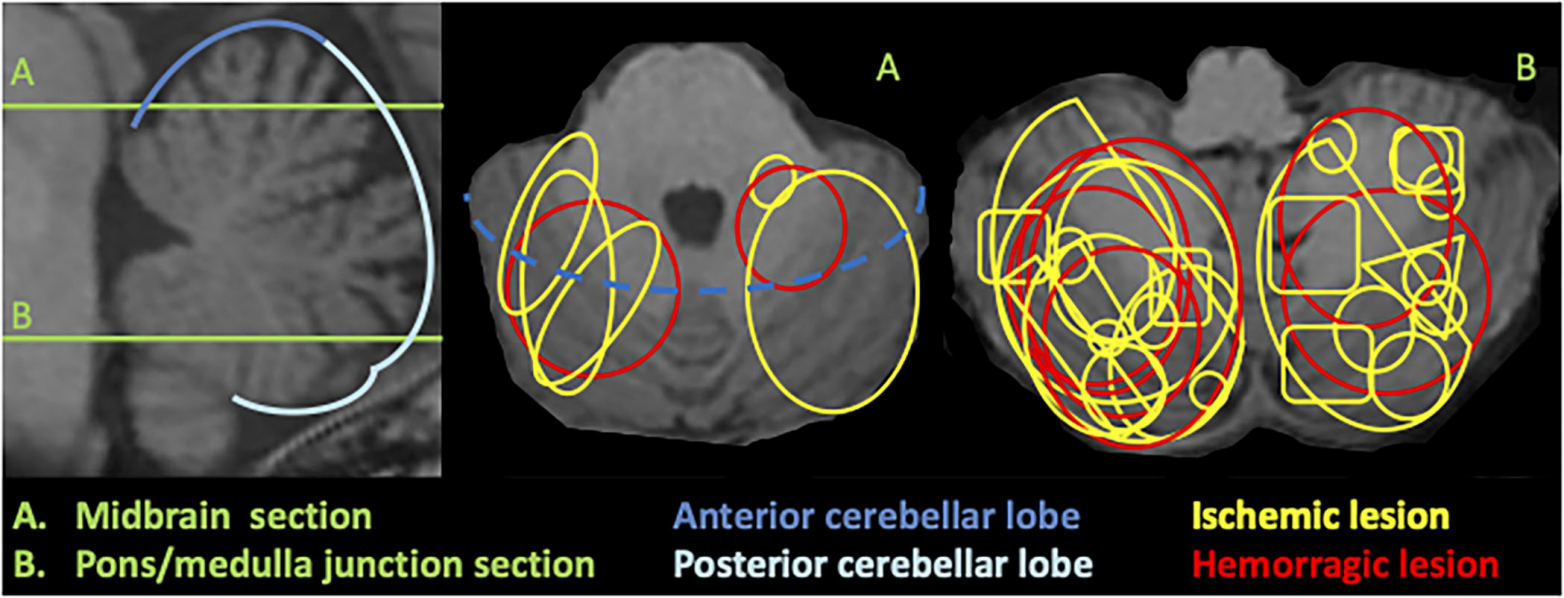
Schematic illustration of cerebellar lesions superimposed on 3DT1 MRI. Sagittal section (left) shows the level of the axial slices (middle and right): **(A)** corresponds to a midbrain level and **(B)** to a pons/medulla junction section.

During the study period, 1,508 patients were admitted to the stroke unit, and 25 of those who presented had an acute vascular cerebellar lesion (1.7%). Patients' characteristics are summarized in Table 1. The mean age was sixty-four, and lesions were ischemic in 18/25 patients and bilateral in 5/25. In ischemic lesions, the posterior inferior cerebellar artery (PICA) territory was most commonly affected, followed by the superior cerebellar artery (SCA) territory. The lesion was located in the posterior cerebellar lobe in all but one patient and predominantly in the right cerebellar hemisphere. Figure 1 illustrates schematically the lesion sizes and locations. The median admission NIHSS score was 1.

**Table 1:** Patients' characteristics.

Age (Years, Mean ± SD)	64 ± 11
Females / Males (*n*)	4 / 21
Cerebellar lesion	
*Ischemic (n)*	18
Unilateral (*n*)	14
Bilateral (*n*)	4
PICA Territory	16
SCA Territory	5
AICA Territory	0
*Hemorragic (n)*	7
Unilateral (*n*)	6
Bilateral (*n*)	1
Cerebellar lesion location	
*Laterality*	
Only Right/ Only left	12/8
Right > Left	3
Left> Right	2
*Affected lobe*	
Anterior lobe only	1
Posterior lobe only	17
Both	7
NIHSS	
Mean ± SD	2.3 ± 2.8
Median [Range]	1 [0–9]

PICA, posterior inferior cerebellar artery; AICA, anterior inferior cerebellar artery; SCA, superior cerebellar artery; NIHSS, National Institute of Health Stroke Scale.

### Cerebellar cognitive affective syndrome

CCAS-Scale was possible to realize in all patients. All patients failed at least one CCAS-S item. Twenty-one patients displayed a definite CCAS (84%), 3/25 (12%) a probable CCAS, and 1/25 (4%) a possible CCAS. Semantic category switching and digit span backward tests were the items most patients failed (18/25, 72% of cases), followed by phonemic fluency and verbal registration tests (17/25, 68% of cases). CCAS-S results are detailed in Table 2.

**Table 2:** CCAS-Scale.

	**Cerebellar stroke (*n* = 25)**	**Number of subjects under passing score, *n* (%)**	**Passing score / Maximum score**
Semantic fluency (number of correct words, mean ± SD)	14.8 ± 7.1	12 (48%)	15/26
Phonemic fluency (number of correct words, mean ± SD)	7.7 ± 4.9	17 (68%)	9/19
Category switching (number of correct alternations, mean ± SD)	6.7 ± 3.8	18 (72%)	9/15
Digit span forward (correct numbers of a serie, mean ± SD)	5.2 ± 1.2	14 (56%)	5/8
Digit span Backward (correct numbers of a serie, mean ± SD)	2.8 ± 1.2	18 (72%)	3/6
Cube drawing (mean score ± SD	10.5 ± 5.1	11 (44%)	11/15
Verbal recall (number of words, mean ± SD)	7.7 ± 4.3	17 (68%)	10/15
Similarities (correct answers, number of words, mean ± SD)	6.2 ± 2.4	10 (41%)	6/8
Go-No Go (mean score ± SD)	1.5 ± 0.8	9 (36%)	0/2
Affect (number of non-affected items, mean ± SD)	4.2 ± 1.8	8 (32%)	4/6
**Total**	**68.2** **±21.3**		**120**
**Failed Items**	**5.2** **±2.12**		**<1**
**Median**	**5 (1–8)**		
**1**	**3**		**1 CCAS possible**
**2**	**1**		**2 CCAS probable**
**≥3**	**21**		**3 CCAS definite**

SD, standard deviation; CCAS, cerebellar cognitive and affective syndrome.

## Discussion

The main findings of this study are that a definite cerebellar cognitive affective syndrome is present in most patients with acute cerebellar vascular lesions and that the CCAS-S can be easily completed at bedside tests in acute stroke settings.

The findings of this study, albeit limited by its monocentric nature and the size of the sample, are likely to be generalizable to other populations of acute cerebellar vascular lesions. In fact, our cohort matches the usual proportion of acute vascular cerebellar lesions in stroke units, ranging between 1.5% (2) and 2.3% (1). Similarly, the clinical characteristics of the reported population of an acute vascular cerebellar lesion are considered in terms of age (1, 2, 14), sex (1, 2, 14), admission NIHSS (3, 4, 28, 29), vascular territory involved (30–32), rate of bilateral lesions (1, 31), and predominant involvement of cerebellar posterior lobes (14). However, a selection bias toward less severe cases in our cohort is possible due to the fact that patients with acute cerebellar vascular lesions who need surgery for acute hydrocephalus or acute brainstem compression are not usually hospitalized in the stroke unit but neurosurgery and intensive care units. Such complications occur in 10 to 20% of acute vascular cerebellar lesions and missed inclusion in our cohort (29, 33).

Since the description of the CCAS in 1998 (22), several studies confirmed that almost all patients with acute cerebellar vascular lesion displayed significant cognitive impairments in a wide range of cognitive domains, corresponding to a CCAS (14, 34–36) that mirrored the characteristics of Schmahmann's seminal report (22). However, the identification of a CCAS in those studies required the use of a full neuropsychological test battery, an assessment that requires over an hour in trained hands and is exhausting for acutely ill patients. Those facts limit the application of a full neuropsychological test battery in acute stroke settings. In contrast, in our cohort, the CCAS-S could be performed in all patients and allowed to screen for a CCAS in 10 min, highlighting its yield in the acute cerebellar stroke context. This report, therefore, brings evidence for the validity of the CCAS-S in acute cerebellar disorders and supports previous findings that relied on the CCAS-S to describe cognitive disorders in degenerative cerebellar diseases such as Friedreich Ataxia (20), SCA3 (37), in a mixed cohort of degenerative cerebellar ataxia (38), as well as in patients with chronic cerebellar stroke (39).

The high rate of CCAS in our cohort, with 24/25 subjects displaying lesions in cerebellar posterior lobes, is consistent with lesion-symptom mapping and functional neuroimaging studies that associate CCAS and cerebellar role in cognition to cerebellar posterior lobes (14, 16, 40). The higher rate of cerebellar right-sided posterior lesions in our cohort may also contribute to a more severe cognitive clinical pattern due to the loss of cross-connections between the dominant hemisphere and the right cerebellum (14, 41). Yet, this association between the right cerebellar lesion and worse cognitive outcomes is inconstant and needs further investigation in larger groups of subjects (35). Compared with patients with cerebellar degenerative diseases or chronic cerebellar stroke, patients with acute cerebellar vascular lesions failed more items [5 compared with 3 in Naeije et al. (20), Maas et al. (37) and Chirino-Pérez et al. (39)] and had a worse CCAS-S total raw score [68/120 against 72/120 in Benussi et al. (42) and 88/120 in Chirino-Pérez et al. (39)]. Such poorer performances in patients with acute cerebellar injury are probably related to the CCAS pathophysiology. In fact, the CCAS is thought to build up from the disconnection of neocortical areas involved in cognitive processes and the cerebellum, corresponding to a cerebellocortical diaschisis (CCD) (20, 24). In degenerative disorders, this diaschisis is gradual and allows compensatory mechanisms as highlighted in degenerative cerebellar ataxias (43–46). At the acute vascular cerebellar lesion stage, both cognitive impairments and CCD on functional brain imaging are maximal (35, 47–49), while compensatory strategies through plasticity or recovery have not yet developed. Over time, cognitive impairments related to cerebellar vascular impairment partially improve, suggesting that the acute disconnection from the cerebellum might recover or be compensated (32, 39). Our patients mostly failed the CCAS-S item relating to verbal fluency, attention, and working memory. Neuroanatomically, verbal fluency is considered to rely more on executive than language functions (50, 51) and is dependent on the prefrontal cortex integrity, similarly to attention (52) and working memory (53). Patients with acute cerebellar vascular lesion failed the item that depends on frontal cortex integrity, which parallels the metabolic brain functional imaging studies on CCD that showed that the frontal cortex was the most metabolically impaired cortical area after a cerebellar lesion (48, 49) and supports CCD as the main pathophysiology for CCAS. Only one-third of patients failed the “affect” item of the CCAS when patients with cerebellar disorders display a much higher rate of non-cognitive psychiatric symptoms and social cognition disorders when formally tested (54–57). The self-reported nature of this item may explain its lack of sensitivity in the CCAS-S. This observation is also made in degenerative cerebellar disorders (20) and may warrant the evolution of the “affect” item in further CCAS-S version. The role of the CCD in CCAS related to cerebellar stroke was also further demonstrated by the long-term consequences of cerebellar vascular lesions (58). In fact, a study from 2021 described an atrophy of the neocortical areas functionally connected to the cerebellum in proportion to the acute cerebellar lesion volume (59).

In summary, this study shows that a CCAS is highly prevalent after acute vascular cerebellar injury and likely reflects acute CCD. This study also positions the CCAS-S as a highly sensitive and practical tool to screen for cerebellar cognitive disorders in the stroke context. Further studies are required to assess the relation between CCAS-S scores at acute and chronic stages and the magnitude of the CCD through both functional and structural brain imaging longitudinal studies.

## Data availability statement

The raw data supporting the conclusions of this article will be made available by the authors, without undue reservation.

## Ethics statement

The studies involving human participants were reviewed and approved by CUB-Hopital Erasme Ethics Committee. Written informed consent for participation was not required for this study in accordance with the national legislation and the institutional requirements.

## Author contributions

NL, AA, and GN: study design, data collection, analysis, and manuscript writing. All authors contributed to the article and approved the submitted version.

## Funding

GN is a Postdoctorate Clinical Master Specialist at the FRS-FNRS (Brussels, Belgium).

## Conflict of interest

The authors declare that the research was conducted in the absence of any commercial or financial relationships that could be construed as a potential conflict of interest.

## Publisher's note

All claims expressed in this article are solely those of the authors and do not necessarily represent those of their affiliated organizations, or those of the publisher, the editors and the reviewers. Any product that may be evaluated in this article, or claim that may be made by its manufacturer, is not guaranteed or endorsed by the publisher.
